# Characterization of reproductive endocrinology and infertility (REI) fellowship applicants: guiding our mentees toward success

**DOI:** 10.1186/s40738-017-0045-x

**Published:** 2017-12-08

**Authors:** Jason Franasiak, Daniel Kaser, Linnea Goodman, George Patounakis, Caroline Juneau, Scott Morin, Shelby Neal, William Schlaff, Richard Scott

**Affiliations:** 1 Sidney Kimmel Medical College at Thomas Jefferson University, Philadelphia, PA 19107 USA; 2 Reproductive Medicine Associates of New Jersey, NJ, USA; 3 University of Central Florida College of Medicine, 6850 Lake Nona Blvd, Orlando, Florida 32827 USA

**Keywords:** Education, Fellowship, OB/GYN, Resident

## Abstract

**Background:**

Advanced subspecialty training in reproductive endocrinology and infertility (REI) entails a competitive application process with many data points considered. It is not known what components weigh more heavily for applicants. Thus, we sought to study the REI fellow applicant and compare 1) those who apply but do not receive an interview, 2) those who receive an interview but do not match, and 3) those who successfully match.

**Methods:**

This retrospective cohort study was conducted at a single REI fellowship program from 2013 to 2017. Academic variables assessed included standardized test scores and total number of publications listed on their *curriculum vitae*. Logistic regression models were constructed to determine variables that were predictive of being offered an interview in our program and of matching in any program.

**Results:**

There were 270 applicants, of which 102 were offered interviews. Interviewed applicants had significantly higher mean USMLE 1 and CREOG scores, as well as total publications and total abstracts. There was no difference in Step 2 and Step 3 scores or in number of book chapters. Of those interviewed, USMLE scores remained predictive of matching in any program; however, publications and scientific abstracts were no longer predictive.

**Conclusions:**

The decision to offer applicants interviews appears to be influenced by objective standardized test scores, as well as a threshold of academic productivity. These items are less predictive of matching once the interview process begins, indicating that other factors, such as performance during the interview day, may be more heavily weighted.

## Background

There has been a focus on attracting and retaining high quality resident physicians in obstetrics and gynecology many of whom comprise and outstanding pool of applicants for post-resident fellowship training [1, 2] Positions in reproductive endocrinology and infertility (REI) training programs are increasingly competitive, and the process for applicant selection is not uniform. The characteristics of highly-ranked residency applicants and the degree to which they subsequent pursue leadership positions have been described in other subspecialties [3, 4], but little data exist to guide the fellow applicant in REI. This lack of data can lead to confusion among applicants about the competitiveness of their application and the likelihood of matching.

Until the 2017 academic cycle, two professional organizations governed the REI fellowship match process. The Society for Reproductive Endocrinology and Infertility (SREI) served as a clearinghouse for applications by receiving the initial standardized applications and coordinating their distribution to programs participating in the match. Subsequently, the National Resident Matching Program (NRMP) served as a centralized place where applicants and programs ranked one another. Thus, those who initially applied through SREI did not always participate in the NRMP match process either because they ultimately elected not to pursue fellowship or, probably more commonly, because they were not invited to interview.

According to the NRMP, the match rate for those who ranked at least one program in the match between 2012 and 2016 was 62.3–68.3% [5]. This match rate would be considerably lower if all those who applied through the SREI application, but were not granted interviews were to be taken into account.

To that end, we sought to characterize the REI fellow applicant and compare 1) those who applied but did not receive an interview in our program, 2) those who receive an interview but do not match in any program, and 3) those who successfully match in any program.

## Methods

All applicant files for the Rutgers-Robert Wood Johnson/Thomas Jefferson fellowship program in REI were reviewed over a five-year period, including applications for fellowship training beginning July 2013 to July 2017. The fellowship transitioned from Rutger-Robert Wood Johnson to Thomas Jefferson in 2017 without change of core facility, faculty, and research core. This retrospective anonymized data review was IRB exempt. Applications were placed into three groups: those who applied but did not receive an interview in our program; those who received an interview in our program but did not match into any fellowship program; and those who ultimately matched into any program. At this fellowship institution, interviews are granted based upon program director and current fellow applicant review.

In reviewing the applicant files, the following data were extracted if provided: medical school training; residency program training; Alpha Omega Alpha (AOA) Honor Society status; United States Medical Licensing Exam (USMLE) scores for Step 1, Step 2 Clinical Knowledge (CK), and Step 3; and scores on the Council on Resident Education in Obstetrics and Gynecology (CREOG) exams for the first, second, and third years of residency.

Academic productivity was likewise assessed, according to the total number of manuscripts (including those in press and progress), the number of published citations in a peer-reviewed journal (i.e., containing a PMID number), the number of book chapter contributions, and finally, the number of scientific abstracts listed for presentation at scientific meetings.

Given that many applicants during the study period exhibited some level of academic productivity, a subanalysis was performed to assess whether a “threshold of productivity” existed. Accordingly, the proportion of applicants having any publications, ≥1 publications and ≥2 publications were compared to those below this threshold.

All statistical analyses were performed using SPSS statistical software (IBM Corp. Armonk, NY). Each variable was assessed for normality utilizing histograms with overlying normality curve and Q-Q plots. Comparison of variables was accomplished with Student’s *t*-test, Mann-Whitney U test, Fisher’s Exact test, and Pearson’s Chi-square test where appropriate. Correlation tests for normally and non-normally distributed values were assessed using Pearson Correlation and Spearman’s Rho tests. A predication model was created based upon the variables which predicted the chance of matching. A *P*-value of <0.05 was considered significant.

## Results

There were 270 applicants during the five-year application period (mean of 54 applicants per year). Of the 270 applicants, there were 211 US medical graduates and 59 foreign medical graduates (FMG); 47.1% of US medical graduates were offered interviews, as compared to only 5.1% of FMG. All FMG applicants offered an interview had trained in US obstetrics and gynecology residency programs.

When looking at total applicants and residency program affiliation, applicants were categorized as belonging to an academic resident program if housed within a university, academic-affiliated program if the training was done at a hospital affiliated with a university, and community program if the training was done at a hospital unaffiliated with a university. In all, 70.4% came from academic residency programs, 9.6% from academic affiliated programs, and 20.0% from community programs.

As for the geographic distribution of applicants, the residency program locations were noted to be in the Northeast, Midwest, South and West as defined by the U.S. Census. The majority of the applicants came from the Northeast which represented 49.3% of applicants. There were 17.4% of applicants from residency programs in the Midwest, 20.4% from the South, and 7.8% from the West Coast. There were 5.1% of those who applied the reported training outside of the United States.

In total there were 102 applicants offered an interview for five fellowship position, one per year over the course of the analysis. The characteristics of those receiving an interview versus those who did not are listed in Table 1. Those fellowship applicants who were offered an interview had significantly higher mean scores on the USMLE 1 and CREOG exams as well as more total publications and total abstracts. When analyzing Step 2 and 3 scores, as well as book chapters written, there were no differences between the groups.

**Table 1:** Characteristics of those who applied and received an interview versus those who did not

	Interview Not Offered (*n* = 168)	Interview Offered (*n* = 102)	*P*-value
USMLE Step 1	219.8 ± 17.8	226.1 ± 19.3	0.043^a^
USMLE Step 2CK	233.3 ± 17.6	238.8 ± 17.0	0.051^a^
USMLE Step 3	215.1 ± 14.6	218.7 ± 13.1	0.130^a^
CREOG Year 1	187.6 ± 16.4	196.2 ± 15.4	0.021^a^
CREOG Year 2	202.7 ± 17.1	210.5 ± 12.7	0.025^a^
CREOG Year 3	210.7 ± 14.7	219.2 ± 13.2	0.009^a^
Total Publications	3 [1–5]	4 [2–6]	0.004^b^
Total Abstracts	4 [2–7]	5 [3–7]	0.018^b^
≥1 Book Chapter	20/168 (12)	16/102 (16)	0.483^c^

Data presented as mean ± standard deviation, median [interquartile range] or n(%)

^a^Student’s T-test

^b^Mann-Whitney U Test

^c^Pearson’s Chi-Square

The unadjusted models predicting the chance of being offered an interview are shown in Table 2. While increased scores on standardized tests were predictive, having any publications and any abstracts increased the chance of obtaining an interview with an OR of 7.2 [95%CI 2.1–24.2] (*p* = 0.001) and 3.1 [95%CI 1.1–8.3] (*p* = 0.028), respectively.

**Table 2:** Unadjusted models for being offered an interview

Variable	OR [95% CI]	P-value
USMLE 1 (per 10 point increase)	1.194 [1.004–1.420]	0.044
USMLE 2CK (per 10 point increase)	1.204 [0.997–1.455]	0.053
CREOG Yr 3 (per 10 point increase)	1.597 [1.096–2.327]	0.015
Total Publications >0 vs 0 (ref)	7.174 [2.129–24.168]	0.001
Total Publications >1 vs < =1 (ref)	3.750 [1.895–7.420]	<0.001
Total Publications >2 vs < =2 (ref)	2.030 [1.199–3.438]	0.008
Total Book Chapters >0	1.377 [0.677–2.798]	NS
Total Abstracts >0 vs 0 (ref)	3.077 [1.131–8.371]	0.028
Total Abstracts >1 vs < =1 (ref)	3.205 [1.425–7.207]	0.005
Total Abstracts >2 vs < =2 (ref)	1.917 [1.085–3.387]	0.025

Of the 102 offered interviews, 87 (85.3%) matched into programs while 15 (14.7%) did not. Demographics of those matching into a fellowship program as compared to those not matching amongst interviewed applicants are listed in Table 3. Here, only USMLE scores were predictive. Similarly, in the unadjusted model, USMLE scores were predictive but others variables were not. These findings are summarized in Table 4.

**Table 3:** Characteristics of those matching to fellowship versus those who interviewed but did not match

	Not Matched	Matched	*P*-value
USMLE Step 1	213.7 ± 16.5	228.3 ± 19.0	0.006^a^
USMLE Step 2CK	224.7 ± 12.7	241.3 ± 16.5	<0.001^a^
USMLE Step 3	212.5 ± 10.2	219.9 ± 13.3	0.052^a^
CREOG Year 1	190.5 ± 14.2	197 ± 15.5	0.335^a^
CREOG Year 2	205.2 ± 14.2	211.4 ± 12.5	0.276^a^
CREOG Year 3	212.7 ± 15.2	220.3 ± 12.7	0.195^a^
Total Publications	4 [2–5]	4 [2–7]	0.508^b^
Total Abstracts	5 [3–7]	5 [3–7]	0.798^b^
At Least 1 Book Chapter	2/15 (13%)	14/87 (16%)	1.000^c^

Data presented as mean ± standard deviation or as median [interquartile range]

^a^Student’s T-test

^b^Mann-Whitney U Test

^c^Fisher’s Exact Test

**Table 4:** Unadjusted models for matching among those interviewed

Variable	OR [95% CI]	P-value
USMLE 1 (per 10 point increase)	1.494 [1.102–2.026]	0.010
USMLE 2CK (per 10 point increase)	1.818 [1.262–2.618]	0.001
CREOG Yr 3 (per point increase)	1.718 [0.756–3.903]	0.196
Total Publications >0 vs 0 (ref)	3.036 [0.258–35.75]	0.337
Total Publications >1 vs < =1 (ref)	1.185 [0.233–6.034]	0.838
Total Publications >2 vs < =2 (ref)	1.313 [0.407–4.237]	0.649
Total Book Chapters >0	1.247 [0.253–6.142]	0.786
Total Abstracts >2 vs < =2 (ref)	0.895 [0.229–3.498]	0.873

Of note, when analyzing the academic productivity in terms of total number of manuscripts, published peer-reviewed manuscripts, book chapters, and scientific abstracts, these were not found to be predictive of matching once granted an interview. When analyzing the “threshold of productivity” for those who interviewed, no defined threshold for scientific abstracts threshold was predictive of matching.

## Discussion

Matching into an REI training program is competitive, and mentoring applicants toward success can be challenging. According to data from the NRMP, the number of programs participating in the match has ranged from 36 to 40 over the period of 2012 to 2016 [5]. Those programs have offered from 42 to 48 positions over the same time period. There were between 60 and 69 applicants in any given year nationwide who participated in the NRMP process from 2012 to 2016. This amounted to 1.4–1.6 applicants per available position and resulted in a match rate of 62.3–68.3% [5]. The data presented by NRMP include only those applicants who create a profile and participate in the matching process, and thus likely include only those who received interview offers at programs and not all of those who initially completed the SREI application. Accordingly, this match rate may overestimate the true value when all applicants (including those who apply, but do not interview) are considered.

This descriptive cohort shows that the typical REI applicant is scientifically productive and scores well on standardized tests. This generalization may not come as a surprise, but the specifics provided by these data can serve as a more concrete directive to potential applicants. For those hoping to be granted an interview, careful preparation in the years prior to application, with dedication to academic performance and standardized testing, is important. At our institution the decision to offer an interview is often based upon objective data such as USMLE scores and CREOG scores. Of note, there also appears to be an emphasis placed on a threshold of academic productivity.

Interestingly, aside from USMLE scores, the objective data gleaned from applications seem to be somewhat less predictive of matching once the interview process begins. This suggests that, once an applicant is granted an interview, that other factors, such as letters of recommendation and interview day performance, may be more heavily weighted in the final ranking process.

There are few published experiences in this realm. Uhlenhake et al. published on the experience of dermatopathology applicants [3]. They noted a lower mean number of publications than seen in our cohort (3.1 versus 4.0), however they say a much larger range of published original research. Of note, this study only looked at the 5 matched fellows in their particular program and they did not have access to or did not analyze all matched fellows, both at their program and at other programs, as was done here.

There are several limitations to these data. They are gleaned from a single program based in the Northeastern region of the US. There may be regional differences in applicants who decide to apply due to geographic preferences. Further, this particular program was established in 2010 and thus is a newer training program. We also only had access to data from applicants who applied to our program, so these results may not be generalizable to all fellowships. Further, during the course of this data collection, the first round of application data was collected and distributed to programs through a centralized application process coordinated by the Society for Reproductive Endocrinology and Infertility (SREI). SREI does not publish the application data like the NRMP. Thus, we are not able to know how many applicants applied nationally and what percentage applied to this program.

Finally, the overall match rate among those who interviewed at our program (85.3% of those who interviewed) was higher than the range from the NRMP (62.3–68.3%) over a similar timeframe. It is possible that these data characterize those fellow applicants more likely to match than the average fellow applicant and should be considered when mentoring future applicants.

As a point for future research, while several of the objective factors are noted to be associated with matching into fellowship, it is not known how these factors predict academic productivity and clinical performance in fellowship and beyond. This would likely require a multi-center analysis of factors and performance and would an interesting area for research extension.

## Conclusion

This observational study provides a five-year single center summary of REI applicants. The field is competitive with only two-thirds of those who receive interviews at programs ultimately matching. The number who apply initially and ultimately match is far lower. This presents a challenge when it comes to mentoring those who desire to enter the field. These data help future applicants compare their academic performance to peers and characterize those who successfully navigate the process versus those who do not.

## Abbreviations

AOA: Alpha Omega Alpha
CREOG: Council on Resident Education in Obstetrics and Gynecology
FMG: Foreign Medical Graduate
NRMP: National Resident Matching Program
PMID: PubMed Identification
REI: Reproductive Endocrinology and Infertility
SREI: Society for Reproductive Endocrinology and Infertility
USMLE: United States Medical Licensing Examination
